# 

**Published:** 1898-05-21

**Authors:** 


					THE HOSPITAL. . '"v " The standard of highest purity."? Lancet, May 21st, 1898.
CADBURY'S ^ ww ^ CADBURY'S
COCOA
ABSOLUTELY PURE. m ^ "j NO DRUGS OR ALKALIES USED
COCOA
jLASnn** IMrc rrnu IsirihuA
7? WICH HOSPITAL
No. 608. Vol. XXIV. [?????] Saiubday,
May 21st, 1898.
[S?b*Sf?0?iMra"'] PBICB TWOPENCE.

				

